# Mental Health Problems Among Children and Adolescents From a Sports Sociology Perspective

**DOI:** 10.2196/60513

**Published:** 2024-07-03

**Authors:** Yuan Li, Qun Zhai, Weihang Peng

**Affiliations:** 1 Faculty of Health Sciences and Sports Macao Polytechnic University Macao Macao; 2 Faculty of Applied Sciences Macao Polytechnic University Macao Macao

**Keywords:** sociology of sport, children, adolescents, mental health, systematic review, intervention, digital technology, parenting, technology, parenting program, engagement, support, adverse childhood experiences

We read the systematic review by Aldridge et al [1] recently published in the *Journal of Medical Internet Research* with interest. First, I would like to commend the author group for their efforts in publishing this article. The topic of the research is not only relevant for the current times but also showcases a high level of academic rigor in research methods and data analysis.

The study makes a particularly prominent contribution to mental health problems among adolescents and adverse experiences among children. The authors effectively provide a comprehensive evaluation of technology-assisted parenting programs through systematic review methods and address the challenges of adverse childhood experiences (ACEs) at the family level. The data analysis section deserves special mention, where the authors used the Stouffer method to combine *P* values, providing preliminary quantitative evidence for the association between specific engagement strategies and engagement outcomes.

Although the article is excellent in multiple aspects, there are some limitations. The heterogeneity in the definition and measurement of engagement and the lack of engagement outcome data were mentioned in the article, which may have limited the depth and breadth of the analysis.

Mental health problems among adolescents are closely related to ACEs. The field of sports sociology can play an important role in addressing this issue. From the perspective of sports sociology, parental involvement plays a crucial role in the development of children. Parents are not only their children's first coaches but also their life mentors, and their behaviors and attitudes have a profound impact on their children's mental health and participation in physical activity [2].

First, parents' support and encouragement can enhance their children's confidence and self-esteem, which is essential for their mental health. Parental involvement can also stimulate their children's interest in physical activity and help them establish positive exercise habits [3,4]. Regular participation in physical activity helps children maintain a healthy weight, prevent chronic diseases, and improve physical fitness. Similarly, positive family interactions and a supportive family environment can promote a child's overall development, including their physical health and social skills [5].

Future research can explore the impact of family physical activity on parenting behavior, how positive interactions among family members can be promoted through physical activity, and how these interactions positively affect the family environment from a sports sociology perspective. Moreover, we can further explore physical activity to reduce factors within the family that may adversely affect child development, such as stress and conflict.

Overall, this article provides us with valuable knowledge and insights that make an important contribution to the development of the field of adolescent mental health problems and childhood adversity experiences. Despite some limitations, these are precisely the areas that future research can continue to explore and expand. By combining theories and methods from the sociology of sports, we can aim to find innovative solutions to current and future challenges.

Thank you for the opportunity to share our thoughts on the mental health problems of children and adolescents from a sports sociology viewpoint.

## Abbreviations

ACE: adverse childhood experience
